# β-Asarone Reverses Chronic Unpredictable Mild Stress-Induced Depression-Like Behavior and Promotes Hippocampal Neurogenesis in Rats

**DOI:** 10.3390/molecules19055634

**Published:** 2014-04-30

**Authors:** Haiying Dong, Zhiying Gao, Hua Rong, Ming Jin, Xiaojie Zhang

**Affiliations:** Department of Pathology, Qiqihar Medical University, 333 BuKui Street, JianHua District, Qiqihar 161006, China

**Keywords:** depression, β-asarone, brain-derived neurotrophic factor, neurogenesis

## Abstract

In this study, we investigated the influence of β-asarone, the major ingredient of *Acorus tatarinowii* Schott, on depressive-like behavior induced by the chronic unpredictable mild stresses (CUMS) paradigm and to clarify the underlying mechanisms. The results show that β-asarone treatment partially reversed the CUMS-induced depression-like behaviors in both the forced swim and sucrose preference tests. The behavioral effects were associated with increased hippocampal neurogenesis indicated by bromodeoxyuridine (BrdU) immunoreactivity. β-Asarone treatment significantly increased the expression of brain-derived neurotrophic factor (BDNF) at levels of transcription and translation. Moreover, CUMS caused significant reduction in ERK1/2 and CREB phosphorylation, both of which were partially attenuated by β-asarone administration. It is important to note that β-asarone treatment had no effect on total levels or phosphorylation state of any of the proteins examined in ERK1/2-CREB pathway in no stress rats, suggesting that β-asarone acts in a stress-dependent manner to block ERK1/2-CREB signaling. We did not observe a complete reversal of depression-like behaviors to control levels by β-asarone. To our knowledge, the present study is the first to demonstrate that adult neurogenesis is involved in the antidepressant-like behavioral effects of β-asarone, suggesting that β-asarone is a promising candidate for the treatment of depression.

## 1. Introduction

Depression is one the most common psychiatric disorders, with a 10%–20% lifetime prevalence [1]. Chronic exposure to stressful life events is an established risk factor for the development of major depression in humans [2]. Although depression has been associated with impaired neurotransmission of serotonergic, noradrenergic, and dopaminergic pathways [3], the monoamine hypothesis no longer provides a satisfactory explanation for the pathophysiology of depression or the mechanism of action of antidepressant drugs [4].

Accumulating evidence has shown alternative mechanisms to elucidate the therapeutic efficacy of antidepressants [5,6]. These include the effect of antidepressants on neurogenesis in adult hippocampus of rodents [7]. Clinical findings showed that hippocampal volume in patients with depression is reduced compared to the volume in healthy people [8,9]. Chronic stress exposure also causes atrophy of neurons in rodent hippocampus [10], which also contributes to decreased volume of hippocampus regions reported in brain imaging studies of major depressive disorder patients [11]. Thus, it has been suggested that depression may be associated with decreased hippocampal plasticity.

Some recent studies have demonstrated that increase of new neurons in the hippocampus after administration of antidepressant agents attenuated behavior changes in the stress-induced models and patients [12,13]. *Post-mortem* studies also demonstrate that brain-derived neurotrophic factor (BDNF) levels are increased in the hippocampus of humans who were taking an antidepressant at the time of death [14]. The behavioral response to antidepressant administration is blocked in conditional BDNF knockout mice [15]. These have led to the proposal of the “neurotrophin hypothesis of depression” [16]. A post-mortem study has also found that levels of extracellular signal-regulated kinases (ERK) activity and expression are decreased in the hippocampus of depressed suicide patients [17]. ERK1/2 phosphorylates cAMP responsive element binding protein (CREB) which governs the transcription of a number of genes including BDNF [18]. The ERK1/2–CREB cascade has been shown to be important for neuronal survival [19].

**Figure 1:**
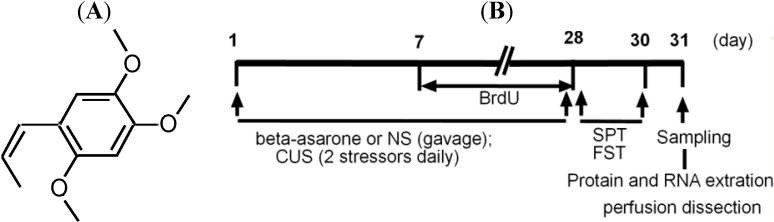
(**A**) Chemical structure of β-asarone and (**B**) protocol used in this study.

Despite a wide range of antidepressants available, 30% to 40% of patients with major depression fail to respond to first-line antidepressant treatment [20], and further highlight a major unmet need for novel and more efficacious antidepressant agents. β-Asarone (for its structure, see Figure 1A) is a major constituent of *Acorus tatarinowii* Schott, which has traditionally been widely used in Chinese medicine for the treatment of depression. β-Asarone easily passes through the blood brain barrier [21]. Several lines of evidence have suggested that β-asarone has neuroprotective effects *in vitro* and *in vivo* [22,23]. The aim of this study was to investigate the hypothesis that interruption of the ERK1/2-CREB signaling pathways by β-asarone might induce hippocampal neurogenesis in chronic unpredictable mild stresses (CUMS)-exposed rats. The results presented in this report support our hypothesis.

## 2. Results and Discussion

### 2.1. β-Asarone Treatment Produces Antidepressant-Like Behavioral Responses in CUMS-Exposed Rats

**Figure 2:**
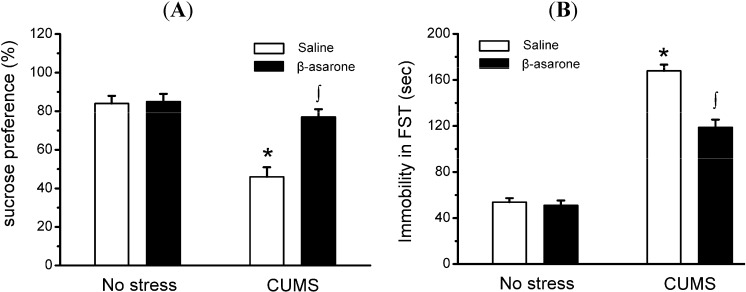
β-Asarone treatment produces antidepressant-like behavioral effects in CUMS-exposed rats. Rats were orally treated with β-asarone (25 mg/kg per day) or saline for 28 days while being exposed to CUMS or control conditions. (**A**) a two-bottle preference test was used to evaluate the sucrose preference, and which were expressed as a ratio of the volume of sucrose solution consumption to the volume of total fluid intake. (**B**) the forced swimming test was used to evaluate the depression-related behavior. Note that CUMS-exposed rats receiving β-asarone displayed decreased immobile time and preference for sucrose, while immobile time and preference for sucrose in β-asarone-treated rats not different from no stress rats. Data were presented as mean ± S.D., *n* = 15 per group. *, *p* < 0.05 compared with no stress/saline animals; ∫, *p* < 0.05 compared with CUMS/saline animals (analysis of variance and SNK *post hoc* test).

The CUMS rat model of depression is a well-established model for detecting antidepressant activity of candidate compounds [24]. References should be in numeral order, please reformat the reference in the main text and references section. SPT showed that level of sucrose preference was reduced by 45% in CUMS-exposed rats as compared with non-stress control rats, which is similar to what Yazir *et al.* have reported earlier [25]. CUMS rats receiving daily oral administration of β-asarone showed a significant 67% increase in sucrose preference compared with the CUMS-exposed rats receiving saline treatment (*p* < 0.05). This is not due to the inability of CMS animals to drink, because the amount of tap water consumption did not decrease throughout the experiments (data not shown). However, β-asarone has no effect on sucrose preference in non-stress control rats (Figure 2A).

As shown by FST (Figure 2B), immobile time was increased by 203% in CUMS-exposed rats as compared with non-stress control rats (*p* < 0.05). Administration of β-asarone to CUMS-exposed rats, but not non-stress control rats, resulted in a significant reduction by 29% in time spent immobile compared with the CUMS-exposed rats (*p* < 0.05). Swim speed was also assessed in FST to determine whether differences in immobility time could be attributed to non- depressive factors. We find that CUMS-exposed rats did not differ during FST from non-stress rats in swim speed (data not shown), indicating that the changes observed in the SPT were specific to this test.

### 2.2. β-Asarone Increases Hippocampal Neurons Neurogenesis in CUMS-Exposed Rats

**Figure 3:**
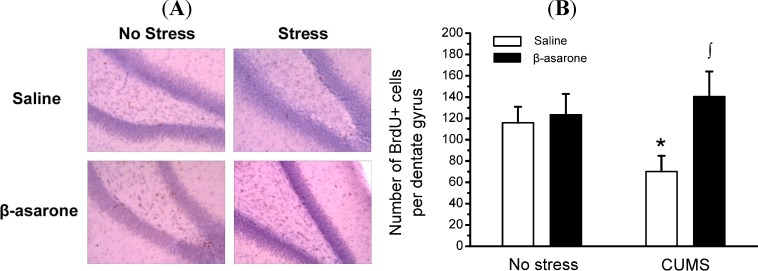
β-Asarone treatment promotes hippocampal neuronal neurogenesis in CUMS-exposed rats. Rats were orally treated with β-asarone (25 mg/kg per day) or saline for 28 days while being exposed to CUMS or control conditions. (**A**) Representative photomicrographs depicting the distribution of newly born cells (*i.e.*, BrdU^+^ cells) in the subgranular zone-granule cell layer of hippocampi from rats. (**B**) Histograms of the number of BrdU-positive cells in the subgranular zone-granule cell layer of hippocampi from rats. Data were presented as mean ± S.D., *n* = 6 per group. *, *p* < 0.05 compared with no stress/saline animals; ∫, *p* < 0.05 compared with CUMS/saline animals (analysis of variance and SNK *post hoc* test).

In order to determine the influence of β-asarone on survival of newborn cells in the adult hippocampus, BrdU was administered after CUMS. Total numbers of BrdU-positive cells that survived for 3 weeks were counted in the hippocampus of rats. Similar to the results observed in a long-term study by Shin and colleague, BrdU positive cells were moderate in the adult hippocampus [26]. We noticed a significant decrease of the number of BrdU-positive cells in CUMS-exposed rats by approximately 39% compared with non-stress control rats (*p* < 0.05). CUMS-exposed rats, but not non-stress control rats, receiving β-asarone displayed a significant increase of BrdU-positive cells in the hippocampus by 100% compared with the CUMS-exposed rats (*p* < 0.05) (Figure 3A,B).

### 2.3. Involvement of ERK1/2 and CREB Phosphorylation in Neuroprotection Effects of β-Asarone in CUMS-Exposed Rats

**Figure 4:**
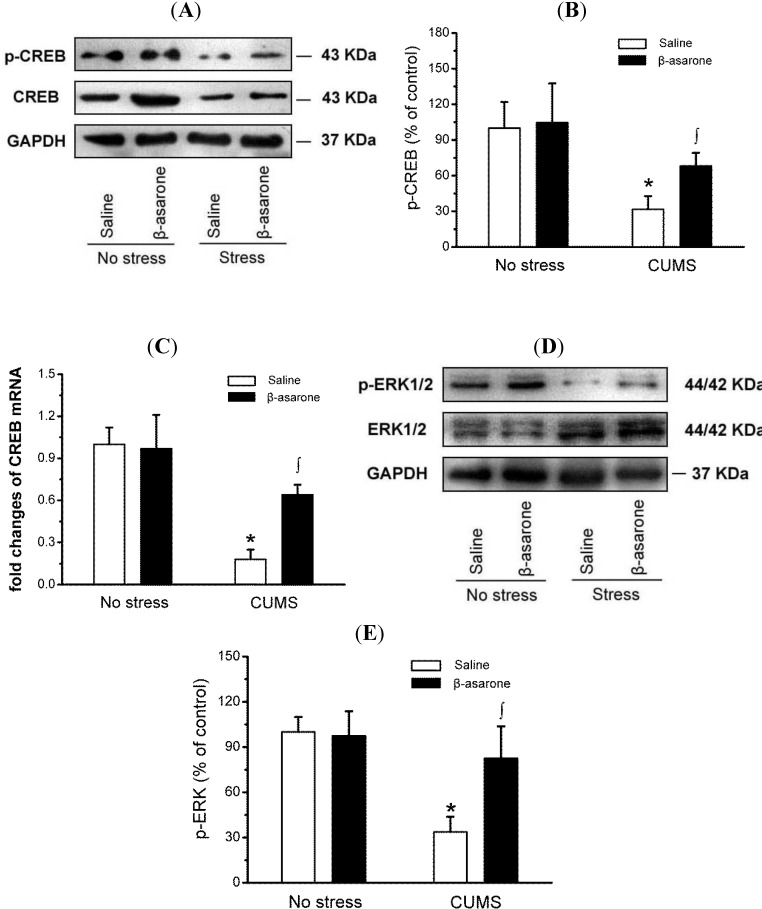
β-Asarone treatment increases ERK1/2 and CREB phosphorylation in hippocampus of CUMS-exposed rats. Rats were orally treated with β-asarone (25 mg/kg per day) or saline for 28 days while being exposed to CUMS or control conditions. (**A**) Phospho-protein levels of CREB were determined by immunoblot analysis with antibody against phosphorylated CREB. Total CREB levels showed equivalent protein loading. A representative immunoblot for p-CREB, CREB and GAPDH. (**B**) The graph demonstrates the densitometric analysis of CREB phosphorylation (normalized to total CREB). Note that the levels of total CREB and GAPDH remained constant. (**C**) Total RNA was isolated from hippocampus using RNAiso reagent and used for cDNA synthesis. The mRNA levels CREB was detected by real-time PCR and the 2^−ΔΔCt^ method. (**D**) Phospho-protein levels of ERK1/2 were determined by immunoblot analysis with antibody against phosphorylated ERK1/2. Total ERK levels showed equivalent protein loading. A representative immunoblot for p-ERK1/2, ERK1/2 and GAPDH. (**E**) The graph demonstrates the densitometric analysis of ERK1/2 phosphorylation (normalized to total ERK1/2). Note that the levels of total ERK1/2 and GAPDH remained constant. Data were presented as mean ± S.D., *n* = 6 per group. *, *p* < 0.05 compared with no stress/saline animals; ∫, *p* < 0.05 compared with CUMS/saline animals (analysis of variance and SNK *post hoc* test).

Accumulating evidence has shown that the ERK1/2-CREB signaling system may be the target for the antidepressant action of antidepressants and participate in the neuronal mechanism of depression [27,28]. Owing to posphorylation of CREB in on Ser133 reflecting activity of CREB, we determined the levels of CREB phosphorylation in CUMS-exposed rats. As shown in Figure 4A,B, western blotting analysis revealed that CUMS-exposed rats exhibited, as expected, a reduction in posphorylation of CREB by approximately 68% compared with the non-stress control rats (*p* < 0.05). However, β-asarone treatment significantly increased CREB phosphorylation by 114% in CUMS-exposed rats (*p* < 0.05), and indicating β-asarone treatment elevating the activity of CREB. Consistent with protein changes, β-asarone treatment also increased mRNA levels of CREB in CUMS-exposed rats (Figure 4C). The activation of CREB by ERK1/2 plays a critical role in the formation of long-lasting neuronal plasticity. Therefore, we further determined effect of β-asarone on phosphorylation of ERK1/2 in CUMS-exposed rats (Figure 4D,E). The phospho-ERK1/2 levels were 70% lower in CUMS-exposed rats compared with the non-stress control rats (*p* < 0.05). Βeta-asarone treatment increased phospho- ERK1/2 levels in CUMS-exposed rats by 133% (*p* < 0.05). The increased levels of ERK1/2 and CREB were not attributable to an increase in total ERK1/2 or total CREB.

### 2.4. β-Asarone Increased BDNF Expression in CUMS-Exposed Rats

**Figure 5:**
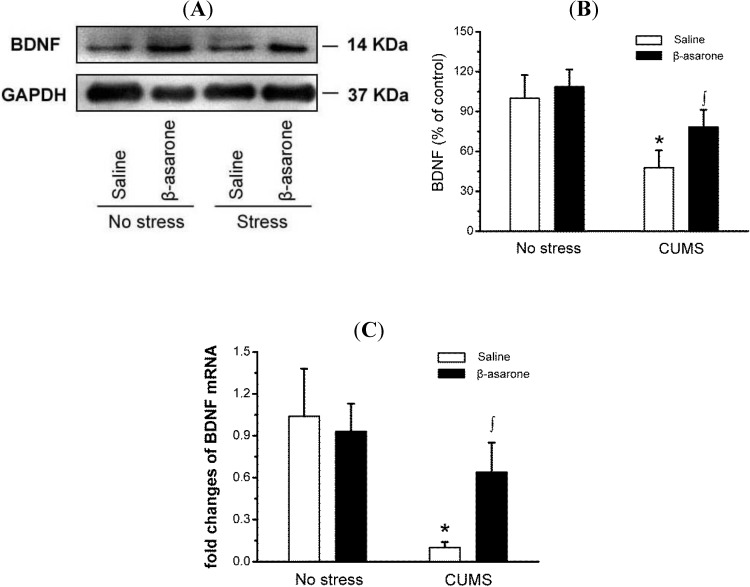
β-Asarone increases BDNF expression in hippocampus of CUMS-exposed rats. Rats were orally treated with β-asarone (25 mg/kg per day) or saline for 28 days while being exposed to CUMS or control conditions. (**A**) BDNF protein levels were determined by immunoblot analysis with antibody against BDNF. GAPDH served as a loading control. A representative immunoblot for BDNF and GAPDH. (**B**) The graph demonstrates the densitometric analysis of BDNF protein expression (normalized to GAPDH). (**C**) Total RNA was isolated from hippocampus using RNAiso reagent and used for cDNA synthesis. The mRNA levels BDNF was detected by real-time PCR and the 2^−ΔΔCt^ method. Data were presented as mean ± S.D., *n* = 6 per group. *, *p* < 0.05 compared with no stress/saline animals; ∫, *p* < 0.05 compared with CUMS/saline animals (analysis of variance and SNK *post hoc* test).

BDNF is a member of neurotrophin family associated with the survival, maintenance, and growth of neurons [29]. Several lines of evidence suggest that BDNF is involved in depression, and the expression of BDNF is reduction in brain of depressed patients [30,31]. As shown in Figure 5A,B, the western blotting results showed that BDNF was 52% less in CUMS-exposed rats than in non-stress control rats (*p* < 0.05). CUMS-exposed rats treated with β-asarone significantly increased the BDNF levels by 63% compared with the CUMS-exposed rats (*p* < 0.05). We further determined effect of β-asarone on the BDNF mRNA levels using quantitative real-time RT-PCR assay. Consistent with the results of protein levels, real-time PCR revealed that β-asarone treatment significantly increase the BDNF mRNA levels (Figure 5C).

### 2.5. Discussion

In this study, we demonstrate for the first time that administration of β-asarone concurrent with CUMS produces antidepressant-like behavioral responses in animal models of depression, as measured both by SPT and FST. We found that hippocampal neurons neurogenesis is almost parallel to antidepressant-like behavioral responses, indicating that neurogenesis contributed to the antidepressant effects of β-asarone. These effects are accompanied by activation of ERK1/2-CREB pathway. The development of depressive behaviors, notably anhedonia, with CUMS exposure is necessary for antidepressant treatment to reverse these effects [32]. CUMS is considered ‘one of the best animal models that capture core symptoms of depression’, although stress and behavioral testing variables also make it one of the most difficult to establish [33]. CUMS satisfies the criteria of predictive validity, face validity, and construct validity as a behavioral model for depression [34]. In order to model CUMS experiences, several different paradigms using rats have been employed (e.g., tail pinch, maternal separation, footshock, social aggression, and restraint) [35]. Chronic unpredictable stress procedure used by this study imitate repeated but mild stressful events. In this report, traditional methods of assessing depression, such as SPT and FST, were performed to determine depression-like behavior induced by CUMS. As shown by SPT, which is commonly used to assess the hedonic drive of animals after chronic mild stress, in the CUMS-exposed rats we observed a level of sucrose preference that a significant decrease compared with unstressed rats, which coincided with the CUMS-induced immobility in FST. Both results suggested that CUMS may be a useful model of human conditioned depression that develops subsequent to chronic stress exposure. The current results are consistent with previous research testing the sensitized effects of stress exposure on depression-like behavior in rodents [36].

*Acorus tatarinowii* Schott has long been used to alleviate depression in the clinical practice of Traditional Chinese Medicine. It has been documented that *Acorus tatarinowii* Schott exerts its principal pharmacological effect on the central nervous system [37]. Wu *et al.* have demonstrated that β-asarone, the major constitutent of this plant, can pass through the blood-brain barrier easily, and the brain is the major organ for distribution of β-asarone after oral administration [21]. In the present study, β-asarone administration produces antidepressant-like behavioral responses in animal models of depression induced by CUMS, which causes behavioral deficits that are similar to those observed in major depressive disorder [38], as indicated by reduce immobility time in FST and increase sucrose preference in SPT. Given the relatively short treatment duration of our study, further studies are required to determine whether or not chronic β-asarone administration can completely counteract the behaviors abnormalities seen in CUMS-exposed rats.

It was originally thought that new neurons are generated in the developmental brain, but some experimental evidence suggesting that neurogenesis occur in the adult brain was presented in the 1960s [39]. During the last decade the scientific insufficiency of the monoaminergic hypothesis of depression became obvious [40]. The most robust link between impaired neuroplasticity and major depressive disorder derives from a large number of studies reporting impaired neurogenesis in subjects displaying depressive-like symptoms [41]. One proposed mechanism for the therapeutic efficacy of antidepressants is the induction of new neurons and recovery of normal function in the adult hippocampus. Suppression of hippocampal neurogenesis abolished the behavioral effects of antidepressants [42]. The hippocampus has long been associated with the modulation of emotional responses [43]. As reported earlier, stress-induced atrophy and loss of hippocampal neurons may contribute to the pathogenesis of depression [10]. The behavioral effects of chronic use of antidepressant drugs may be mediated by the stimulation of neurogenesis in the hippocampus of adult rats [44]. BrdU labeling is widely used in neurogenesis-related studies, as BrdU, which is an analogue of thymidine, is rapidly incorporated into dividing and proliferating cells [45]. In this study, we found that a β-asarone treatment significantly attenuated the CUMS-induced decrease in the number of BrdU-positive cells in the hippocampus, which suggests that oral administration of β-asarone for 28 days increases hippocampal cell proliferation. Our results may confirm further the antidepressive-like effects of cell proliferation-inducing drugs.

ERK1/2 is one of the mitogen-activated protein kinases involved in numerous cellular processes, including long-term neuronal plasticity and survival. Two major isoforms of ERK, ERK1 and ERK2, are very similar in sequence and widely expressed in neurons. It has been well documented that ERK1/2 signaling events involved in synaptic plasticity [46]. Promoters of many neuronal genes containing CRE sequence is activated in response to various signaling transduction cascades [47]. ERK1/2 is upstream to CREB in the control of neurogenesis [48]. Our study demonstrated that β-asarone activated ERK1/2 and CREB in the hippocampus although the causal relationship remains unclear *in vivo*. CREB-mediated transcription involves gene changes responsible for the synaptic plasticity that includes BDNF [49]. Both CREB and BDNF are downstream targets of the ERK pathway.

Inadequate neurotrophic support in adult individuals could ultimately be an underlying mechanism leading to decreased capacity of brain to adaptive changes and increased vulnerability to neurotoxic damage [50]. BDNF is not only a putative target of the action of antidepressant drugs but produces itself an antidepressant-like effect, and may thus be one of the molecular mediators of antidepressant drugs [51]. Recent evidence suggests antidepressant drugs treatment increase the expression of neurotrophin BDNF. Such observations suggest depression and the antidepressant effect implicate BDNF signaling as a mediator of neuroplasticity. BDNF is a mediator involved in neuronal survival and plasticity of neurons in the hippocampus [52]. Several clinical studies on major depressive disorder have shown that blood BDNF is associated with depression response [53]. *Post-mortem* studies have shown that BDNF levels are much higher in the brains of patients with depression who had received pharmacotherapy [54]. Our study demonstrated that BDNF protein expression in the hippocampus was decreased in CUMS-exposed rats. On the contrary, after treatment with β-asarone, the both the protein and mRNA expression of BDNF protein were, at least in part, reversed. Moreover, β-asarone has no effect on BDNF in non-stress control rats. So, CUMS exposure may render the BDNF more responsive to β-asarone. Whether that responsiveness is due to hippocampal neurons damage caused by CUMS is not known, but this type of pathological responsiveness to β-asarone may also contribute to its antidepressant-like activity. The results suggests that effects of β-asarone may be milder in healthy brain and that β-asarone treatment may affect only disturbed cellular functions and cause relatively few adverse effects. In addition, Pearson’s correlation coefficients revealed p-ERK1/2, p-CREB, and BDNF expression in hippocampus of rats was significantly correlated with CUMS-induced depression-like behavior (data not shown).

However, the possibility that other unrelated protein kinases were responsible for antidepressant-like behavioral effects of β-asarone cannot be entirely excluded based on the current results. These findings are consistent with previous data from the literature indicating that ERK1/CREB pathway represents a key target of various neurogenesis agents, including antidepressant drugs [27].

## 3. Experimental Section

### 3.1. Animals

Male Sprague–Dawley rats (Vital River Laboratories, Beijing, China) weighing 225–250 g at the onset of experiments were housed three per cage in a temperature-controlled environment with a 12 h light/dark cycle, and free access to food and tap water. All experimental protocols were approved by the Animal Care and Use Committee of Qiqihar Medical University. All experimental procedures were performed during the light cycle.

### 3.2. CUMS Procedure

Rats were subjected for 3 weeks to a variable sequence of mild, unpredictable stressors which cause depressive-like behavioral changes [55]. A total of 10 different stressors were used (two stressors per day). The stressors used were placement in a 4 °C environment, lights off for 3 h (11 a.m.–2 p.m.), lights on overnight, strobe light overnight, rotation on a shaker, aversive odor, food and water deprivation, 45 °C tilted cages, crowding and isolation.

### 3.3. β-Asarone Preparation and Treatment Protocols

β-Asarone was prepared as described previously [56]. Briefly, root of *Acorus tatarinowii* Schott was purchased from a local market in Qiqihar, and dried. The essential oil of *Acorus tatarinowii* Schott was extracted by classic hydrodistillation. β-Asarone was purified from the essential oil by freezing crystallization. The administration of β-asarone (98% purity by HPLC) was initiated concurrently with CUMS. CUMS-exposed rats were administered intragastrically β-asarone (25 mg/kg/day), or an equivalent volume of saline as control for 28 consecutive days in a volume of 0.01 mL/g body weight (for experimental schedules, see Figure 1B). The dose of β-asarone was established in pilot studies.

### 3.4. Sucrose Preference Test (SPT)

Animals were habituated to a sucrose solution for 48 h (starting day 28), followed by both food and water were removed the night before the sucrose test. Animals were then allowed to drink sucrose solution or tap water. Sucrose and water consumption were determined by measuring the change in volume of fluid. Sucrose preference was defined as percentage of consumed sucrose solution of the total amount of liquid drunk during the 1-h test.

### 3.5. Forced Swim Test (FST)

On day 29, rats were placed in a glass cylinder (30 cm diameter) filled to a depth of 45 cm with water for 10 min, followed on day 30 (24 h later) by a 5 min test. The time spent immobile during the swim session was recorded by an investigator blinded to the treatment that rats received. Swimming was defined as movement around the tank. A rat is judged to be immobile when it remains passively floating in the water in a hunched position, its nose just above the water.

### 3.6. BrdU Labeling and Immunohistochemical Staining

BrdU (5-bromo-2'-deoxyuridine, Sigma, St Louis, MO, USA, 25 mg/kg, i.p.) dissolved in 0.007 N NaOH in 0.9% NaCl was intraperitoneally injected once daily for 21 days. Rats were anesthetized and transcardially perfusion-fixed with ice-cold 4% paraformaldehyde in phosphate-buffered saline. Brains were removed, and post-fixed overnight. Tissues were then submerged in 30% sucrose for 2 days for cryoprotection and sectioned on a freezing microtome at 10 μm. Sections were first incubated in 2 mol/L HCl for 30 min at 37 °C. After quenching endogenous peroxidases with H_2_O_2_ in methanol, tissues were blocked by non-immune goat serum for 20 min. Tissue slides were incubated at 4 °C overnight with rat anti-BrdU antibody (1:200; Cell Signaling Technology Inc., Beverly, MA, USA); washed in PBS; then incubated with horseradish peroxidase–conjugated immunoglobulin G (Maxin Biotechnology, Fuzhou, China) and Streptomyces avidin peroxidase (Maxin Biotechnology). The peroxidase reaction was visualized by using 3,3'-diaminobenzidine tetrahydrochloride as chromagen (Maxin Biotechnology). Sections were counterstained with hematoxylin. Incubation without the primary antibody was performed as a control for the background staining. Mumbers of BrdU-positive cells in the dentate gyrus on 3 different sections per animal, six animals per group, were counted manually by a researchers, blind to the experimental procedures, using an inverted fluorescence microscope under the 10× microscope objective.

### 3.7. RNA Isolation and Real-Time Polymerase Chain Reaction (PCR)

Total RNA was isolated from hippocampal tissue using TRIzol reagent (CWBIO, Beijing, China). First-strand cDNA was synthesized from 2–3 μg of total RNA using SuperScript II reverse transcriptase (Invitrogen, Carlsbad, CA, USA) and oligo(dT)18 primers following the manufacturer’s instructions. Real-time PCR were performed by using SYBR^®^ Premix Ex Taq™ kit (Takara Biotechnology, Dalian, China) in an ABI7300 real-time PCR system (Applied Biosystems, Foster City, CA, USA). Primers used were as follows: CREB sense, 5'- ACA GAT TGC CAC ATT AGC -3'; CREB antisense, 5'- CTC CTC CCT GGG TAA TGG -3'; BDNF sense, 5'- CCC TTC TAC ACT TTA CCT CTTG -3'; BDNF antisense, 5'- GTT TCA CCC TTT CCA CTC CTA -3'; GAPDH sense primer, 5'- GAC AAC TTT GGC ATC GTG GA -3'; and GAPDH antisense 5'- ATG CAG GGA TGA TGT TCT GG -3'. Ct (threshold cycle) data were collected using the Sequence Detection Software version 1.2.3 (Applied Biosystems). The relative quantification of gene expression was analysed by the 2^−ΔΔCt^ method [57]:

Fold change = 2^−ΔΔCt^, where ΔΔCt = (Ct _target gene_ − Ct _GAPDH_) − (Ct _control_ − Ct _GAPDH_)

### 3.8. Western Blot

Cytoplasm proteins were extracted from hippocampus tissues using Cytoplasmic Protein Extraction kit (Beyotime Biotechnology, Haimen, China). The protein concentration was measured using the BCA protein assay kit (CWBIO). Proteins (30 µg) were separated by SDS-PAGE, and transferred to a nitrocellulose membrane for immunoblotting. The membrane was first blocked by incubation in nonfat milk at room temperature for 2 h, and then incubated with antibodies against p-ERK1/2 (1:1,000; Cell Signaling Technology Inc.), ERk1/2 (1:2,000; Cell Signaling Technology Inc.), p-CREB (1:1,000; Cell Signaling Technology Inc.), CREB (1:1,000; Cell Signaling Technology Inc.), BDNF (1:200, Santa Cruz Biotechnology, Santa Cruz, CA, USA), and GAPDH (1:3,000; CWBIO). Immunoreactive bands were then detected by incubating with conjugates of anti-rabbit IgG with horseradish peroxidase and enhanced chemiluminescence reagents (1:4,000; CWBIO) as recommended by the manufacturer. The stripped blots were blocked for 1 h and incubated with primary antibody directed against glyceraldehydes 3-phosphate dehydrogenase (GAPDH) (Santa Cruz Biotechnology) for loading control. Densitometric analysis of immunoreactivity for each protein was conducted using an AlphaImager™ 2200 using the SpotDenso function of AlphaEaseFC™ Software version 3.1.2 (Witec, Littau, Switzerland). Immunoreactivity was normalized to the control group for each protein.

### 3.9. Statistical Analysis

Two-way ANOVA with Bonferonni’s post hoc test was used for the determination of statistical significance. Correlations between neurochemical and behavioral parameters were determined with Pearson's correlation coefficients. Significance level was set at *p* < 0.05. For statistical analysis, the SPSS software package was used (SPSS Inc., Chicago, IL, USA).

## 4. Conclusions

Our results are consistent with the hypothesis that β-asarone produces antidepressant-like behavioral responses, at least in part, by induction of hippocampal neurogenesis when taken orally in CUMS-exposed rats. The effects are associated with the activation of ERK1/CREB signaling pathway, results in up-regulation of the levels of BDNF. Its potent antidepressant activity *in vivo* will encourage clinical application of β-asarone as an antidepressant candidate compound to induce both hippocampal neurogenesis and antidepressant-like effects.
